# The impact of emphysema on surgical outcomes of early-stage lung cancer: a retrospective study

**DOI:** 10.1186/s12890-019-0839-1

**Published:** 2019-04-04

**Authors:** Seijiro Sato, Masaya Nakamura, Yuki Shimizu, Tatsuya Goto, Terumoto Koike, Hiroyuki Ishikawa, Masanori Tsuchida

**Affiliations:** 1 Division of Thoracic and Cardiovascular Surgery, Niigata University Graduate School of Medical and Dental Sciences, 1-757 Asahimachi-dori, Chuo-ku, Niigata-shi, Niigata, 951-8510 Japan; 2 Department of Radiology and Radiation Oncology, Niigata University Graduate School of Medical and Dental Sciences, Niigata, Japan

**Keywords:** Lung cancer, Emphysema, Pulmonary resection, Prognosis, Postoperative complications

## Abstract

**Background:**

The presence of emphysema on computed tomography (CT) is associated with an increased frequency of lung cancer, but the postoperative outcomes of patients with pulmonary emphysema are not well known. The objective of this study was to investigate the association between the extent of emphysema and long-term outcomes, as well as mortality and postoperative complications, in early-stage lung cancer patients after pulmonary resection.

**Methods:**

The clinical records of 566 consecutive lung cancer patients who underwent pulmonary resection in our department were retrospectively reviewed. Among these, the data sets of 364 pathological stage I patients were available. The associations between the extent of lung emphysema and long-term outcomes and postoperative complications were investigated. Emphysema was assessed on the basis of semiquantitative CT. Surgery-related complications of Grade ≥ II according to the Clavien-Dindo classification were included in this study.

**Results:**

Emphysema was present in 63 patients. The overall survival and relapse-free survival of the non-emphysema and emphysema groups at 5 years were 89.0 and 61.3% (*P* < 0.001), respectively, and 81.0 and 51.7%, respectively (*P* < 0.001). On multivariate analysis, significant prognostic factors were emphysema, higher smoking index, and higher histologic grade (*p* < 0.05). Significant risk factors for poor recurrence-free survival were emphysema, higher smoking index, higher histologic grade, and presence of pleural invasion (*P* < 0.05). Regarding Grade ≥ II postoperative complications, pneumonia and supraventricular tachycardia were more frequent in the emphysema group than in the non-emphysema group (*P* = 0.003 and *P* = 0.021, respectively).

**Conclusion:**

The presence of emphysema affects the long-term outcomes and the development of postoperative complications in early-stage lung cancer patients.

## Background

Lung cancer is the most common malignancy and the leading cause of cancer-related death in men worldwide [1]. Tobacco smoking is the most common risk factor for developing not only lung cancer, but also chronic obstructive pulmonary disease (COPD) [2, 3]. It has been shown that oxidants in cigarette smoke cause chronic biological damage, including DNA injury [4–6], which then results in a predisposition to these pulmonary diseases.

A mixture of small airway disease and emphysema causes airflow limitation in COPD [7]. Pulmonary emphysema is defined as abnormal permanent enlargement of the airspaces distal to the terminal bronchioles accompanied by destruction of their walls without obvious fibrosis [8]. A previous study showed that the severity of emphysema did not reflect the COPD disease stage well [9]. Some severe COPD patients also have terrible emphysema, whereas there are other patients with very little evidence of emphysema. Therefore, one cannot easily classify the COPD phenotypes, such as “pink puffers” and “blue bloaters”, using the severity of emphysema as a criterion. Thus, to assess emphysematous change in the lung and its implications for lung cancer is meaningful. Recent reports have shown that the extent of emphysema on computed tomography (CT) is associated with an increased frequency of lung cancer [10, 11], but the postoperative outcomes of pulmonary emphysema have not been studied in depth [12–14].

Therefore, this study was conducted to investigate the associations between the extent of emphysema detected semiquantitatively on CT scans and long-term outcomes, as well as mortality and postoperative complications, of stage I lung cancer patients after pulmonary resection.

## Methods

### Patients

Of 566 patients with non-small cell lung cancer (NSCLC) admitted from 2009 to 2015 to the Division of Thoracic and Cardiovascular Surgery at Niigata University Hospital, 392 had pathological stage I disease. Because the purpose of this study was to identify whether the extent of emphysema could be a prognostic factor, the exclusion criteria were: 1) neoadjuvant therapy (chemotherapy and/or radiation therapy); 2) the presence of interstitial pneumonia (IP); and 3) incomplete resection with macroscopic or microscopic residual disease. After exclusion of 28 cases, a consecutive series of 364 of these patients who had pathological stage I disease and had undergone lung resection were retrospectively reviewed in the present study. The institutional review board approved this study (Niigata University, 2018–0133) and waived the requirement for informed consent because the study was a retrospective review.

The preoperative patient data included age, sex, body mass index (BMI), smoking habit, spirometric variables, tumor size, surgical procedure (lobectomy or sublobar resection), and the extent of emphysema. The patients with lung cancer located within the outer one-third of the lung field and diameter of ≤2 cm as measured on CT underwent intentional sublobar resection, especially segmentectomy. However, the patients with compromised pulmonary reserve underwent wedge resection. Pulmonary emphysema was diagnosed by visual semiquantitative CT, as described later. Spirometric variables included forced vital capacity (FVC) and forced expiratory volume in 1 s (FEV1). Pathologic data obtained after surgery included histologic subtype, histologic grade, and pleural invasion. The tumors were classified histologically as adenocarcinoma, squamous cell carcinoma, and others and graded as well, moderately, or poorly differentiated carcinoma according to the WHO classification [15]. Pathological stage was determined according to the 7th edition of the TNM classification for lung cancer proposed by the International Association for the Study of Lung Cancer (IASLC) [16].

#### Diagnosis of emphysema

CT images, 1 to 2-mm-thick, were obtained using window levels appropriate for lung (level, − 910 Hounsfield units (HU); width, 1500 HU) from the apex to the diaphragm at full inspiration with the patient in the supine position. All CT scans at 1-cm intervals from each case were coded and masked for identifiable information and then evaluated by one thoracic radiologist (H. I.) and two thoracic surgeons (S.S. and Y.S.) who were blinded to the clinical data.

The system used to score the extent of emphysema on the CT scans was adapted from prior work by Goddard et al. [17] and Bergin et al. [18]. A brief summary was as follows. Each slice was assessed individually, and the right and left lungs were graded separately according to the percentage area that demonstrated changes suggestive of emphysema. Areas of low attenuation and vascular disruption were considered suggestive of emphysema. A grade of 0 was given if there was no abnormality. If less than 25% of the pulmonary parenchyma in a slice was considered to show vascular disruption and low attenuation compared with remaining lung parenchyma, the score was 1; between 25 and 50%, the score was 2; between 50 and 75%, the score was 3; and if more than 75% of the lung parenchyma on 1 side suggested emphysema, the maximum score of 4 was given. This gave a maximum possible of 4 for the right lung and 4 for the left lung or a total of 8 for 1 slice. All slices from the apex to the diaphragm at 1-cm intervals were assessed in each patient (14 to 29 slices per patient). The maximal possible score in a patient in whom 20 slices were taken was 8 × 20 = 160. The final score for each patient was calculated as a percentage of the maximal possible CT score.

The percentage of voxels with attenuation values lower than − 910 HU among the total number of voxels in the entire lung was considered to be the low attenuation area (LAA) [19, 20]. Emphysema was defined as being present if LAAs occupied more than 10% of the lung, according to past reports [11, 12].

#### Postoperative complications

Surgery-related complications were graded according to the Clavien-Dindo classification [21, 22] as follows: grade 0, no complications; grade I, some deviation from the normal postoperative course; grade II, complications requiring management not exceeding intravenous medications, total parenteral nutrition, enteral nutrition, or blood transfusion; grade III, complications requiring surgical, endoscopic, or radiologic intervention; grade IV, life-threatening complications requiring intensive care; and grade V, complications causing the death of the patient. Complications of Grade ≥ II were included in this study.

#### Postoperative follow-up

After the patients were discharged from hospital, they were followed in our outpatient clinic at intervals of 3 months during the first 5 years postoperatively and then every 6 months. During follow-up, at intervals of 6 to 12 months, the patients were routinely evaluated for tumor recurrence or metastasis by chest CT and, if necessary, head and abdominal CT and positron emission tomography with 18F-fluoro-2-d-glucose/computed tomography (FDG-PET/CT).

#### Statistical analysis

The characteristics of the patients are shown as counts and proportions. The categorical variables were compared using the chi-squared test or Fisher’s exact test when there were ≤ 5 observations in a cohort. The unpaired Student’s *t*-test was used to compare quantitative parameters. The relationship between two continuous variables was tested by linear regression analysis. A univariate Cox regression model was used for survival analysis. Using a threshold of *P* < 0.05 on univariate analysis, variables were then entered in a stepwise Cox regression analysis to identify independent factors, with the criterion for retention of factors in the final model being *P* = 0.05. Recurrence-free survival (RFS) was defined as the time from surgery to documented clinical recurrence or death, with overall survival (OS) defined as the time from surgery to death. The Kaplan-Meier method was used to prepare survival curves, which were then compared using the log-rank test. Differences with a *P* value < 0.05 were considered significant. SPSS for Windows Version 25.0 (SPSS, Inc., Chicago, IL, USA).

## Results

### Perioperative patient characteristics

**Fig. 1:**
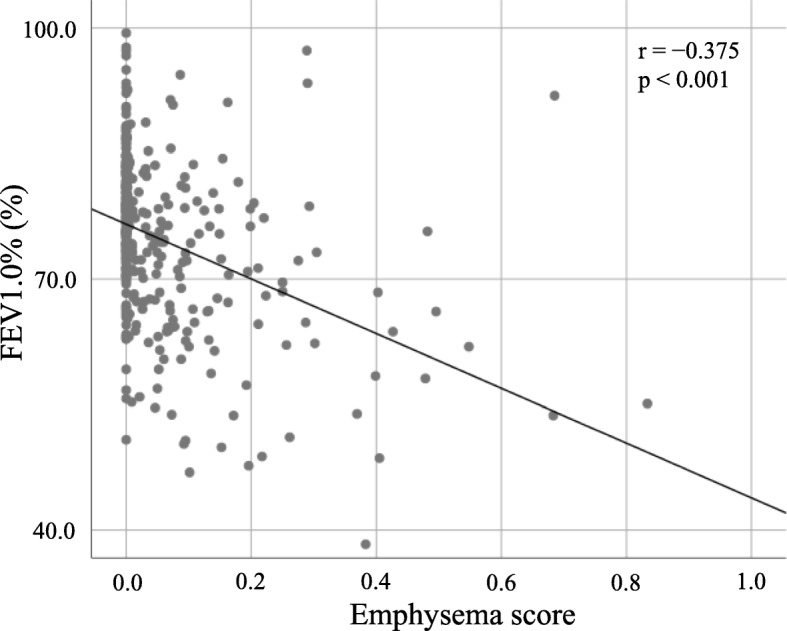
Linear relationship between FEV1/FVC and the percent low attenuation area (%LAA)

Emphysema was diagnosed in 63 (17.3%) and COPD was diagnosed in 94 (25.8%) of the 364 patients. Perioperative patient characteristics are summarized in Table 1. The median follow-up period was 45.8 months (range, 2.4–105 months). The following were more frequent in the emphysema group than in the non-emphysema group: male sex, thinner patients, higher smoking index, squamous cell carcinoma, pleural invasion-positive, and higher histologic grade. On pulmonary function testing, FEV1/FVC was significantly worse, but VC was significantly better in the emphysema group than in the non-emphysema group. Figure 1 shows the linear relationship between FEV1/FVC and %LAA; a weak relationship was noted between these parameters (r = 0.351, *P* < 0.001).

**Table 1:** Patient demographics

Variable^a^	Non-emphysema (*n* = 301)	Emphysema (*n* = 63)	*P*
Sex			
Male	169 (56.1)	54 (85.7)	< 0.001
Female	132 (43.9)	9 (14.3)	
Age (y)	69.0 ± 8.2	71.2 ± 8.0	0.049
BMI (kg/m^2^)	22.3 ± 2.9	21.2 ± 2.9	0.006
Smoking index	565.4 ± 650.4	1305.4 ± 719.1	< 0.001
VC (%)	105.0 ± 62.0	101.7 ± 18.1	0.633
FEV1/FVC (%)	76.2 ± 8.6	67.6 ± 12.9	< 0.001
COPD			< 0.001
No	243 (80.7)	27 (42.9)	
Yes	58 (19.3)	36 (57.1)	
Surgical procedure			0.798
Sublobar resection	119 (39.5)	26 (41.3)	
Lobectomy	182 (60.5)	37 (58.7)	
Tumor size (mm)	22.6 ± 9.8	25.9 ± 11.5	0.038
Histology			< 0.001
Adenocarcinoma	250 (83.0)	33 (52.4)	
Squamous cell carcinoma	37 (12.3)	26 (41.3)	
Other	14 (4.6)	4 (6.3)	
Histologic grade			0.001
Well differentiated	159 (52.8)	18 (28.6)	
Moderately differentiated	107 (35.6)	31 (49.2)	
Poorly differentiated	35 (11.6)	14 (22.2)	
Pleural invasion			< 0.001
Absent	226 (75.1)	33 (52.4)	
Present	75 (24.9)	30 (47.6)	

*BMI* Body mass index, *VC* vital capacity, *FEV1* forced expiratory volume in 1 s; *FVC* forced vital capacity

^a^Categorical data are expressed as numbers, and continuous data are expressed as means ± standard deviation

### Survival analysis

**Fig. 2:**
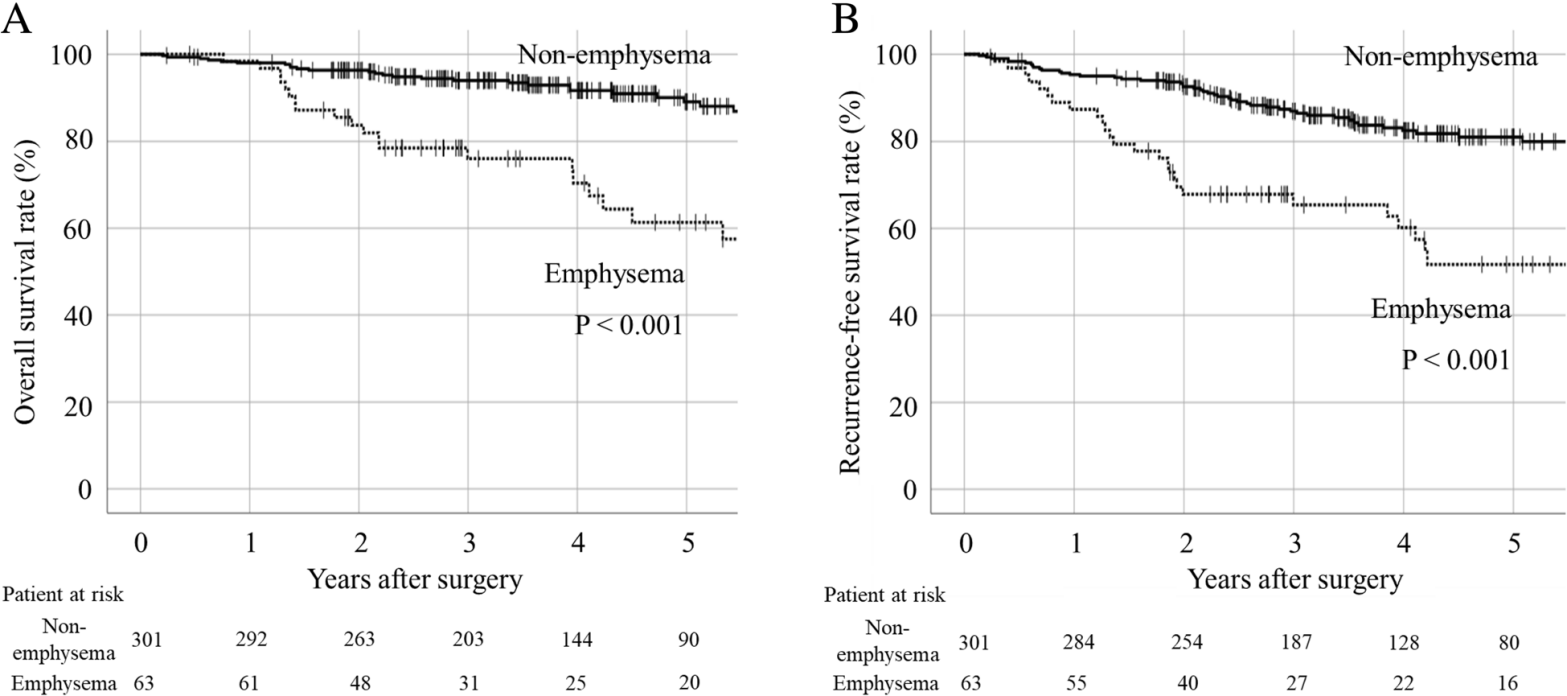
Postoperative overall survival (**a**) and recurrence-free survival (**b**) curves for stage I lung cancer patients with and without emphysema

During the study period, there were 32 deaths (10.6% of patients) in the non-emphysema group and 20 deaths (31.7% of patients) in the emphysema group. Primary lung cancer recurrence was seen in 36 patients (12.0%) in the non-emphysema group and 18 patients (28.6%) in the emphysema group. Five-year overall survival was 89.0 and 61.3% in the non-emphysema and emphysema groups, respectively (*P* < 0.001; Fig. 2a), with 5-year relapse-free survivals of 81.0 and 51.7%, respectively (*P* < 0.001; Fig. 2b).

### Univariate and multivariate analyses of risk factors for long-term surgical outcomes

Using the univariate Cox regression model, male sex, higher age, lower BMI, higher smoking index, lower %VC, lower FEV1/FVC, presence of COPD, histology, higher histologic grade, presence of pleural invasion, and the presence of emphysema were significantly associated with overall survival (Table 2), whereas male sex, higher smoking index, lower %VC, lower FEV1/FVC, presence of COPD, tumor size, histology, higher histologic grade, presence of pleural invasion, and the presence of emphysema were significantly associated with relapse-free survival (Table 3).

**Table 2:** Univariate and multivariate analyses of risk factors for overall survival using a proportional hazard model

		Univariate analysis			Multivariate analysis		
Variable^a^		HR	95% CI	*P*	HR	95% CI	*P*
Sex	Male	3.383	1.648–6.945	0.001			
	Female	1					
Age		1.050	1.013–1.088	0.007			
BMI		0.904	0.819–0.997	0.044			
Smoking Index		1.001	1.000–1.001	< 0.001	1.000	1.000–1.001	0.013
VC (%)		0.98	0.963–0.997	0.021			
FEV1/FVC (%)		0.957	0.932–0.982	0.001			
COPD	No	1					
	Yes	2.332	1.348–4.034	0.002			
Surgical procedure	Sublobar resection	1					
	Lobectomy	1.042	0.599–1.814	0.884			
Tumor size		1.251	0.981–1.596	0.071			
Histology	Adenocarcinoma	1					
	Squamous cell carcinoma	2.529	1.347–4.746	0.004			
	Other	3.159	1.228–8.125	0.017			
Histologic grade	Well differentiated	1			1		
	Moderately differentiated	3.002	1.530–5.888	0.001	2.205	1.115–4.360	0.023
	Poorly differentiated	4.805	2.309–9.998	< 0.001	2.990	1.388–6.442	0.005
Pleural invasion	Absent	1					
	Present	2.107	1.219–3.641	0.008			
Emphysema	Absent	1			1		
	Present	3.263	1.865–5.709	< 0.001	1.927	1.050–3.537	0.034

*HR* hazard ratio, *CI* confidence interval, *BMI* body mass index, *VC* vital capacity, *FEV1* forced expiratory volume in 1 s; *FVC* forced vital capacity

^a^Categorical data are expressed as numbers, and continuous data are expressed as means ± standard deviation

**Table 3:** Univariate and multivariate analyses of risk factors for recurrence-free survival using a proportional hazard model

		Univariate analysis			Multivariate analysis		
Variable^a^		HR	95% CI	*P*	HR	95% CI	*P*
Sex	Male	2.171	1.293–3.647	0.002			
	Female	1					
Age		1.028	0.998–1.058	0.065			
BMI		0.944	0.871–1.	0.155			
Smoking Index		1.001	1.000–1.001	< 0.001	1.000	1.000–1.001	0.006
VC (%)		0.984	0.970–0.998	0.029	0.985	0.971–0.999	0.032
FEV1/FVC (%)		0.965	0.945–0.986	0.001			
COPD	No	1					
	Yes	1.806	1.134–2.874	0.013			
Surgical procedure	Sublobar resection	1					
	Lobectomy	0.940	0.598–1.476	0.787			
Tumor size		1.288	1.054–1.575	0.013			
Histology	Adenocarcinoma	1					
	Squamous cell carcinoma	2.034	1.199–3.450	0.008			
	Other	3.146	1.426–6.942	0.005			
Histologic grade	Well differentiated	1			1		
	Moderately differentiated	2.800	1.647–4.760	< 0.001	1.934	1.111–3.367	0.020
	Poorly differentiated	3.605	1.933–6.722	< 0.001	2.026	1.048–3.915	0.036
Pleural invasion	Absent	1			1		
	Present	2.713	1.734–4.246	< 0.001	1.845	1.154–2.951	0.011
Emphysema	Absent	1			1		
	Present	2.807	1.749–4.504	< 0.001	2.122	1.173–3.839	0.013

*HR* hazard ratio, *CI* confidence interval, *BMI* body mass index, *VC* vital capacity, *FEV1* forced expiratory volume in 1 s; *FVC* forced vital capacity

^a^Categorical data are expressed as numbers, and continuous data are expressed as means ± standard deviation

Using the stepwise Cox regression analysis, the presence of emphysema [hazard ratio (HR), 1.927; 95% confidence interval (95% CI), 1.050–3.537; *P* = 0.034], higher smoking index [HR, 1.000; 95% CI, 1.000–1.001; *P* = 0.013], and higher histologic grade [moderately differentiated: HR, 2.205; 95% CI, 1.115–4.360; *P* = 0.023, and poorly differentiated: HR, 2.990; 95% CI, 1.388–6.442; *P* = 0.005, respectively] were considered independent predictors of poor overall survival (Table 2), whereas the presence of emphysema [HR, 2.122; 95% CI, 1.173–3.829; *P* = 0.013], higher smoking index [HR, 1.000; 95% CI, 1.000–1.001; *P* = 0.006], lower %VC [HR, 0.985; 95% CI, 0.971–0.999; *P* = 0.032], higher histologic grade [moderately differentiated: HR, 1.934; 95% CI, 1.111–3.367; *P* = 0.020, and poorly differentiated: HR, 2.026; 95% CI, 1.048–3.915; *P* = 0.036, respectively], and presence of pleural invasion [HR, 1.845; 95% CI, 1.154–2.951; *P* = 0.011] were considered independent predictors of poor recurrence-free survival (Table 3).

### Postoperative morbidity and mortality

Table 4 summarizes postoperative grade ≥ II cardiopulmonary complications and mortality. The emphysema group had significantly more episodes of pneumonia requiring antibiotic therapy (*P* = 0.003) and of supraventricular tachycardia requiring medication (*P* = 0.021) than the non-emphysema group**.** No patients underwent mechanical ventilation and died within 30 days after surgery, but one patient died of pneumonia during the hospital stay in the non-emphysema group.

**Table 4:** Cardiopulmonary complications and mortality following lung cancer resection

Complication	Non-emphysema (n = 301)	Emphysema (n = 63)	*P*
Pulmonary fistula	26 (8.6)	9 (14.3)	0.165
Bronchial fistula	0 (0)	1 (1.6)	0.173
Pneumonia	6 (1.8)	7 (11.1)	0.003
Emphysema	2 (0.7)	0 (0)	0.683
Arrhythmia	8 (2.7)	6 (9.5)	0.021
30-day mortality	0 (0)	0 (0)	
In-hospital death	1 (0.3)	0 (0)	

## Discussion

In this study, emphysematous lung was found to be the critical predictor of long-term survival in stage I lung cancer patients undergoing pulmonary resection. Furthermore, lung cancer patients with emphysema developed more postoperative cardiopulmonary complications requiring treatment, such as pneumonia and supraventricular tachycardia, than those without emphysema.

The extent of abnormal inflammatory responses in small airways and parenchymal destruction of the lungs promotes airflow limitation in COPD [7]. Several studies have shown that COPD diagnosed on the basis of functional limitation by spirometry worsened the prognosis of early-stage neoplasms [23–27]. However, the clinicopathological characteristics and postoperative outcomes of early-stage lung cancer patients with pulmonary emphysema have not well been studied [13].

With regard to the characteristics of patients with emphysema, some studies have addressed this point. Patients with emphysematous lungs had a higher prevalence of male sex and a smoking history [12, 14]. Regarding histology, emphysema lung was associated with a risk of small cell lung carcinoma (SCLC) and squamous cell carcinoma [28, 29]. In the present study, male sex, thinner patients, higher smoking index, squamous cell carcinoma, high histologic grade, and pleural invasion-positive were more frequent in patients with emphysema, similar to previous reports.

In this study, the correlation between FEV1/FVC and %LAA was found to be significant but weak overall (r = 0.351, *P* < 0.001). This finding was consistent with that of previous studies [9, 30–32], which suggested that the cause of airflow limitation in COPD was not mainly pulmonary emphysema.

In the present study, 5-year overall survival and relapse-free survival rates were 61.3 and 51.7%, respectively. Patients with emphysema in pathological stage I had poorer OS and RFS than those without emphysema. Ueda et al. defined emphysema as the presence of LAAs that occupied more than 5% of the lung based on objective quantification using CT densitometry and reported 5-year OS and RFS rates of 39.4 and 44.0% after pulmonary resection in lung cancer patients with emphysema [13]. In the present study, emphysema was defined as the presence of LAAs that occupied more than 10%, because this was thought to reflect emphysema more strongly. Nevertheless, the reason for the better results of the present study might be due to differences in patient background characteristics. Ueda et al. recruited patients who were all smokers, and 28 (28%) had pathologic stage II or higher [13]. In contrast, the present study included a consecutive series of 364 patients who underwent lung resection for pathological stage I lung cancer. In addition, a few studies investigated the outcomes of lung cancer with emphysema. Zulueta et al. showed that the presence of emphysema was a significant predictor of death from lung cancer, and as for the extent of emphysema, marked emphysema, in which discrete areas of decreased attenuation could be identified in more than one-half of the lung parenchyma, was an independent risk factor for death from lung cancer [33]. Gullon et al. showed that emphysema, which occupied > 10% of the lung, was an independent prognostic factor, but not COPD, in patients with advanced-stage lung cancer [12].

The mechanism of the association between emphysema and a poor prognosis in lung cancer patients has not been fully clarified. Both emphysema and lung cancer are affected by reactive oxidant species caused by tobacco smoking, which induce chronic inflammatory changes in the lungs [34, 35]. Chronic inflammation, through the production of inflammatory cells and inflammatory mediators, including chemokines, cytokines, prostaglandins, and so on, facilitates the survival and proliferation of malignant cells and promotes angiogenesis and metastasis [36]. Various inflammatory mediators, such as tumor necrosis factor-α (TNF-α), transforming growth factor β (TGF-β), interleukin-1 (IL-1), and IL-6, have been reported to promote circulating tumor cell survival and the epithelial-mesenchymal transition, which play an important role in cellular proliferation, migration, invasion and immunosurveillance of NSCLC [37, 38]. Lourenco et al. reported that the extent of emphysema impair the mucociliary function, so carcinogens tend to pool in the emphysematous lung with impaired mucociliary clearance, leading to lung cancer development [39]. Genetically, Yang et al. showed that the patients who carry an α_1_-antitrypsin deficiency allele may have an increased risk for developing lung squamous cell or bronchoalveolar carcinoma [40]. The protein that cannot inhibit neutrophil elastase are regularly polymerized by the α_1_-antitrypsin deficiency. Under the α_1_-antitrypsin deficiency, these protein polymers are chemotactic for neutrophils and produce the inflammatory change in the lung, and lead to early-onset and emphysema [41]. Muller et al. showed that the percentage of fibroblasts positive for senescence-associated β-galactosidase was significantly higher in emphysematous lung than in non-emphysematous lung [42]. They suggested that lung fibroblasts in emphysematous lung were associated with premature aging and therefore predisposed to cancer.

The postoperative complications graded according to the Clavien-Dindo classification in the present study showed that emphysema lung was closely associated with pneumonia and supraventricular tachycardia. Previous studies have suggested that postoperative pulmonary complications were linked to several preoperative variables including obesity [43], COPD [26, 44], smoking habit [43], and so on. Takahashi et al. showed that emphysema based on high-resolution CT (HRCT) was independently associated with pulmonary complications after single lobectomy [45]. Several studies reported that emphysema, bronchiectasis, and bronchial wall thickness on HRCT are predictors of COPD severity [46, 47]. It has been assumed that as COPD is impaired from the alveolar surface to the capillary endothelium region, a decrease in gas exchange space and capillary area occurs. Then, atelectasis due to increased sputum and altered mucociliary clearance also occurs more frequently in patients with emphysema [48].

The risk of supraventricular tachycardia (SVT) following pulmonary resection has been reported in several papers, with an incidence ranging between 10 and 28% [49–51]. In patients with emphysema lung, 6 (9.5%) of 63 had postoperative SVT, and this incidence rate was lower than in previous reports, but only patients with postoperative SVT grade ≥ 2 could be included in the present study. It has remained unclear why SVT develops more frequently in patients with emphysema. A previous study reported that the decrease of elastic recoil induced by emphysema produces a decrease in maximal expiratory airflow and an increase in residual volume, contributing to shortness of breath during exertion and at rest [52]. Emphysema is associated with pulmonary hypertension not only at rest, but also during exercise in patients compared with general subjects [53]. Thus, right heart strain following right ventricular dilatation could occur with an increase in pulmonary artery pressures. Therefore, patients with emphysema might have a higher incidence of SVT than those without emphysema. However, pulmonary artery pressures have not been routinely examined using Swan-Ganz catheters or echocardiograms in our patients, and further study will be required to address this issue.

Our study has several strengths. First, a detailed visual score of the extent of emphysema could be obtained because CT images were viewed from the apex to the diaphragm at 1-cm intervals in each patient. Thus, CT images of 14 to 29 slices were assessed for each patient. Second, postoperative complications were assessed objectively based on the Clavien-Dindo classification. Only complications of Grade ≥ II, which required management, were included in this study. Thus, the subjects with postoperative complications seemed to represent clinically relevant patients in this study. Third, surgical outcomes were reported based on the extent of emphysema in patients with early-stage lung cancer, but so far there have been few reports about this issue [13].

### Limitations

This study has several limitations. First, it was a retrospective, single-institute study with a small sample size. In particular, of the 63 patients in the emphysema group, there were only 9 women. Thus, it would be difficult to generalize these results to all patients, especially women with emphysema. Second, although quantitative standardized assessment with computer algorithms could be used, visual assessment of emphysema based on CT images, which is a subjective task known to be prone to observer variability [54, 55], was also used to assess the presence and extent of emphysema. However, previous studies have shown the validity of this approach [28, 56]. The visual emphysema score has been described as highly correlated with objective volume-based computerized assessment of the whole lung [9]. Third, this study did not evaluate the outcomes of all patients with resected stage I lung cancer because patients with interstitial pneumonia (IP) and combined pulmonary fibrosis and emphysema were excluded. Although Li et al. suggested that the visual emphysema score could distinguish the lesion of emphysema from UIP more accurately than objective volume-based computerized assessment [28], it has been difficult to accurately distinguish emphysema lung from interstitial pneumonia lung. Fourth, the number of patients with advanced lung cancer was too small in our database to make any definitive conclusions about the effect of emphysema. Thus, whether the impact of emphysema on cancer behavior was strong only in early-stage lung cancer remains unknown. To clarify this issue, a larger series of patients with advanced-stage lung cancer would be required.

## Conclusions

The extent of emphysema affects long-term outcomes and the development of postoperative cardiopulmonary complications in surgically resected early-stage lung cancer patients. Thus, it is possible that assessment of emphysema in lung cancer patients using CT before therapeutic intervention could be meaningful to decide the appropriate treatment strategy, such as surgery (lobectomy or sublobar resection), radiotherapy, and so on.

## Abbreviations

BMI: Body mass index
CI: Confidence interval
COPD: Chronic obstructive pulmonary disease
CT: Computed tomography
FDG-PET/CT: Positron emission tomography with 18F-fluoro-2-d-glucose/computed tomography
FEV1: Forced expiratory volume in 1 s
FVC: Forced vital capacity
HR: Hazard ratio
HRCT: High-resolution computed tomography
HU: Hounsfield units
IL: Interleukin
IP: Interstitial pneumonia
LAA: Low attenuation area
NSCLC: Non-small cell lung cancer
OS: Overall survival
RFS: Recurrence-free survival
SCLC: Small cell lung carcinoma
SVT: Supraventricular tachycardia
UIP: Usual interstitial pneumonia
